# Elevated expression of *SLC34A2* inhibits the viability and invasion of A549 cells

**DOI:** 10.3892/mmr.2014.2376

**Published:** 2014-07-14

**Authors:** WEIHAN YANG, YU WANG, QIANG PU, SUJUAN YE, QINGPING MA, JIANG REN, GUOXING ZHONG, LUNXU LIU, WEN ZHU

**Affiliations:** 1 State Key Laboratory of Biotherapy and Cancer Center, West China Hospital, Sichuan University, Chengdu, Sichuan 610041, P.R. China; 2 Department of Thoracic Surgery, West China Hospital, Sichuan University, Chengdu, Sichuan 610041, P.R. China

**Keywords:** complement, initiation and progression, molecular mechanism, A549 lung adenocarcinoma cells, *SLC34A2*, viability and invasion

## Abstract

Abnormal expression of solute carrier family 34 (sodium phosphate), member 2 (*SLC34A2*) in the lung may induce abnormal alveolar type II (AT II) cells to transform into lung adenocarcinoma cells, and may also be important in biological process of lung adenocarcinoma. However, at present, the effects and molecular mechanisms of *SLC34A2* in the initiation and progression of lung cancer remain to be elucidated. To the best of our knowledge, the present study revealed for the first time that the expression levels of *SLC34A2* were downregulated in the A549 and H1299 lung adenocarcinoma cell lines. Further investigation demonstrated that the elevated expression of *SLC34A2* in A549 cells was able to significantly inhibit cell viability and invasion *in vitro*. In addition, 10 upregulated genes between the A549-P-S cell line stably expressing *SLC34A2* and the control cell line A549-P were identified by microarray analysis and quantitative polymerase chain reaction, including seven tumor suppressor genes and three complement genes. Furthermore, the upregulation of complement gene C3 and complement 4B preproprotein (C4b) in A549-P-S cells was confirmed by ELISA analysis and was identified to be correlated with recovering Pi absorption in A549 cells by the phosphomolybdic acid method by enhancing the expression of *SLC34A2*. Therefore, it was hypothesized that the mechanisms underlying the effect of SLC34A2 on A549 cells might be associated with the activation of the complement alternative pathway (C3 and C4b) and upregulation of the expression of selenium binding protein 1, thioredoxin-interacting protein, PDZK1-interacting protein 1 and dual specificity protein phosphatase 6. Downregulation of *SLC34A2* may primarily cause abnormal AT II cells to escape from complement-associated immunosurveillance and abnormally express certain tumor-suppressor genes inducing AT II cells to develop into lung adenocarcinoma. The present study further elucidated the effects and mechanisms of *SLC34A2* in the generation and development of lung cancer.

## Introduction

As the incidence of lung adenocarcinoma has rapidly increased, it has become a major pathological type of lung cancer (1). Revealing the molecular pathogenesis of lung adenocarcinoma may enable improvement in the diagnosis and treatment of this disease.

The gene solute carrier family 34 (sodium phosphate), member 2 (*SLC34A2*) is a member of the SLC34 family located on chromosome 4p15–p16. The full-length cDNA of *SLC34A2* is 4,167 bp with an open reading frame that encodes a 689-amino-acid protein. The *SLC34A2* gene encodes the type 2b sodium-phosphate cotransporter NaPi-IIb (2,3), which is responsible for the transcellular absorption of Pi in an apical membrane (4–6). According to previous studies, mutations in *SLC34A2* led to the occurrence of pulmonary alveolar microlithiasis, testicular microlithiasis and hypophosphatemia (7–9). Previous studies have suggested that the tumorigenesis of several types of cancer might be associated with abnormal expression of *SLC34A2*, including papillary thyroid, ovarian and breast cancer (10).

In 1999, Xu *et al* (6) revealed that *SLC34A2* was expressed in numerous human tissues, with adult and fetal lungs demonstrating the highest levels of expression. Shibasaki *et al* (11) confirmed that targeted deletion of the *SLC34A2* gene resulted in early embryonic lethality, and suggested that *SLC34A2* was a vital gene in early embryonic development. Simultaneously, a study by Kopantzev *et al* (12) confirmed that the mRNA expression level of *SLC34A2* was increased during human lung embryogenesis; however, was decreased in non-small cell lung carcinoma (NSCLC). These studies proposed that *SLC34A2* might be a novel candidate for a molecular marker of NSCLC. It is widely accepted that the decreased expression of a gene in lung cancer tends to exhibit a monotonically increased expression during lung development. By contrast, upregulated genes in various types of lung cancer tend to exhibit a monotonically downregulated expression during lung development (13–15). For example, a key gene of lung embryogenesis (16,17), caveolin 1 (*CAV1*), which has the opposite trend of expression between embryogenesis and tumorigenesis (15), has been implicated in oncogenic cell transformation, tumorigenesis and metastasis (18). Furthermore, downregulation of CAV1 has been confirmed to be a promoting factor in the development of lung cancer (19,20). These studies prompted us to hypothesize that *SLC34A2* might be linked to the onset of lung cancer, and further motivated the investigation of the effects and molecular mechanisms of *SLC34A2* in the initiation and progression of lung cancer.

In the lung, *SLC34A2* is expressed primarily in alveolar type II (AT II) cells (21). The AT II cells are not only responsible for the production of surfactant fluids, but are also the potential pulmonary alveolar epithelium stem cells, which are able to differentiate into alveolar type I (AT I) cells and is capable of self renewal (21–23). Previous studies demonstrated that the AT II cells were a progenitor cell of lung adenocarcinoma and bronchioloalveolar carcinoma (24,25). In addition, Kitinya *et al* (26) and Gazdar *et al* (27) also found that AT II cells might be the progenitor cells of several types of lung carcinoma, including large cell carcinoma, adenocarcinoma and squamous cell carcinoma, particularly lung adenocarcinoma. In addition, previous studies verified that long-term exposure to carcinogenic factors was able to cause AT II cells to transform into lung cancer cells (28,29). In 2009, Xu *et al* (30) found that a diet low in Pi might affect normal lung development by disturbing the Akt-FGF-2 signals associated with tumor progression. Xu *et al* also indicated that pulmonary NaPi-IIb was critical in Pi metabolism. These studies highlighted that a lack of Pi might be associated with the pathogenesis of lung cancer. Thus, it was hypothesized that a lower expression of *SLC34A2* in AT II cells might lead to the deficiency in Pi, which might cause the hyperproliferation and lack of differentiation of AT II cells, and then cause these abnormal AT II cells to transform into lung adenocarcinoma. *SLC34A2* might therefore be important in the development of lung adenocarcinoma.

To examine this hypothesis, the expression of *SLC34A2* in A549 and H1299 lung adenocarcinoma cells compared with normal human bronchial epithelial (HBE) cells was first detected by quantitative polymerase chain reaction (qPCR). The AT II cell-like A549 human lung adenocarcinoma cell line was then selected for further identification of the biological functions of *SLC34A2* in lung cancer cells. The present study preliminarily revealed the effects and mechanisms of *SLC34A2* against A549 lung adenocarcinoma cells *in vitro*, and provided insights into the effects and mechanisms of *SLC34A2* in the generation and development of lung cancer.

## Materials and methods

### Cell culture

The HBE human bronchial epithelial cell line obtained from the American Type Culture Collection (ATCC, Arlington, VA, USA) was cultured in Dulbecco’s modified Eagle’s medium supplemented with 10% fetal bovine serum (FBS; Gibco-BRL, Carlsbad, CA, USA). The cells from the primary explants in their first passage were infected with the recombinant retrovirus LXSN16E6E7 containing the human papilloma virus E6E7 gene. The cells were selected in the presence of 0.4 mg/ml G418 (31). The A549 human lung adenocarcinoma cell line (32) and H1299 cell line obtained from the ATCC were maintained in RPMI-1640 supplemented with 10% FBS. For all experiments described, the cells were incubated in the aforementioned medium at 5% CO_2_ and at 37°C.

### Plasmid construction

According to the *SLCS4A2* full-length coding region (Gene Bank serial no. NM_006424), the sense primer, containing a *Bam*HI site at its 5′-end (SLCS4A2 forward: 5′-GCGGATCCTAATGGCTCCCTGGCCTGAAT-3′) and an antisense primer, containing an *Eco*RI site at its 5′-end (reverse: 5′-GCGAATTCCTACAAGGCCGTGCATTCG-3′) were used to clone the coding DNA sequence of *SLCS4A2* from the pcmv-sport6-*SLCS4A2* (Open Biosystems, Inc., Huntsville, AL, USA) using a PrimeSTAR HS PCR kit (Takara, Dalian, China). The cloned cDNA sequence was connected to the pcDNA3.1 plasmid vector (InvivoGen, San Diego, CA, USA) using a DNA Ligation kit Ver. 2.0 (Takara). Pure pcDNA3.1 and pcDNA3.1-*SLC34A2* plasmids were prepared using an Endofree^TM^ Plasmid Giga kit (Qiagen, Chatsworth, CA, USA) for the following experiments.

### SLC34A2 gene expression analysis by qPCR

Total RNA from cultured cells was isolated using TRIzol reagent (Invitrogen Life Technologies) according to the manufacturer’s instructions. Synthesis of cDNA with reverse transcriptase was performed using a PrimeScript RT reagent kit (Perfect Real Time) (Takara). For *SLC34A2* gene expression analysis, qPCR analysis was performed in the iCycler iQ5 real-time PCR detection system (Bio-Rad Laboratories, Hercules, CA, USA) with SYBR-Green reagents (Bio-Rad Laboratories), *SLC34A2*-specific primers and a probe (Invitrogen Life Technologies). The human GAPDH primer and probe reagents were used as the normalization control in subsequent quantitative analysis.

### Stable transfection of A549 cells with SLC34A2

A549 cells were seeded on 6-well plates at 2×10^5^/well. When the cell density reached 60–70%, they were transfected with the pcDNA3.1-*SLC34A2* and pcDNA3.1 plasmids using Lipofectamine 2000 (InvivoGen). Following 48 h, the transfected cells were replaced with a medium containing G418 (800 μg/ml; Sigma, St. Louis, MO, USA) to eliminate nontransfected cells. Individual colonies appeared following 2 weeks. Then, G418-resistant colonies were isolated into a 96-well plate to be expanded. Following 2–3 weeks, when the quantity of positive transfected cells was enough, they were cultured in 6-well plates with 500 μg/ml G418. Following this, the positive transfected cells were identified by determining whether *SLC34A2* was expressed stably by qPCR and western blot analysis. Stable transfectants were maintained in the medium containing G418 (500 μg/ml). A549 cells stably expressing pcDNA3.1-*SLC34A2* and pcDNA3.1 were designated A549-pcDNA3.1-*SLC34A2* (A549-P-S) and A549-pcDNA3.1 (A549-P), respectively, for further analysis.

### Growth curve

The cells were plated into 96-well plates at 2.0×10^4^ cells/well. The effect of *SLC34A2* on the cell viability of A549 cells was determined using an 3-(4,5-dimethylthiazol-2-yl)-2,5-diphenyltetrazolium bromide (MTT) assay. At the indicated time points (1, 2, 3, 4, 5, 6 and 7 days), the culture medium was replaced with 180 μl of fresh medium, and 20 μl MTT solution (5 mg/ml; Sigma) was added to the culture medium and incubated for another 4 h at 37°C. Following 4 h, the unreacted dye was removed by aspiration. Blue formazan crystals were observed in the well when examined under a microscope (Olympus IX51; Olympus, Tokyo, Japan). Following this, 150 ml dimethylsulfoxide was added to each well and then incubated on a shaker for 10 min at room temperature to dissolve the blue crystal. The absorbance was measured using a microplate reader (Bio-Rad Laboratories) at a wavelength of 490 nm. The percentage cell viability was then calculated using the following formula: [optical density (OD)_490_ (treated cells) / OD_490_ (control)] × 100.

### Colony formation assay

The cells were seeded into 6-well plates in triplicates at a density of 300 cells/well in 2 ml of medium containing 10% FBS. The cultures were regularly replaced with fresh medium in a 37°C humidified atmosphere containing 5% CO_2_ and grown for 3 weeks. The cell clones were fixed with pure methanol and stained for 15 min with a solution containing 0.05% crystal violet, then followed by three rinses with double distilled water to remove excess dye. The colony numbers were counted using a gel documentation system (ImagePro Plus software; Media Cybernetics, Rockville, MD, USA). Additionally, the colony formation rates were calculated in terms of the number of A549-P-S cells and A549-P cells relative to A549 cells.

### Cell invasion analysis

The cell invasion assay was performed using a Boyden chamber (Millipore, Billerica, MA, USA) with BD Matrigel™ (BD Biosciences, Franklin Lakes, NJ, USA). The filters in the upper compartment were loaded with 400 μl serum-free RPMI-1640 containing 5×10^4^ cells, and filters in the lower compartment were filled with 600 μl RPMI-1640 containing 10% FBS. The chamber was then cultured in 5% CO_2_ at 37°C for 24 h. Then, the Matrigel and cells in the upper chamber were removed, and the attached cells in the lower section were fixed with pure methanol and stained with 0.05% crystal violet. The number of migrated cells was counted in five randomly selected power fields (magnification, ×200) under a light microscope. The invasion rates were calculated in terms of the number of A549-P-S cells and A549-P cells relative to A549 cells.

### Microarray analysis

Total RNA extraction from A549-P and A549-P-S using TRIzol reagent (Invitrogen Life Technologies, Gaithersburg, MD, USA) and purification of the RNA using a NucleoSpin^®^ RNA clean-up kit (Macherey-Nagel, Düren, Germany) were completed and prepared for microarray analysis. The commercially available 35K Human Genome Array, including 25,000 human genes, was obtained from CapitalBio Corporation (Beijing, China). Double-stranded cDNAs were synthesized from 1 μg total RNA using the CbcScript reverse transcriptase with the T7 Oligo (dT). The dsDNA was transcribed into cRNA *in vitro* transcription reactions at 37°C for 4–14 h using a T7 enzyme mix. Next, 2 μg of cRNA was reverse transcribed to generate cDNA using CbcScript II reverse transcriptase. The cDNA was fluorescently labeled by Cy5 or Cy3 CPT with the Klenow enzyme following reverse transcription. The cDNA of A549-P-S cells was labeled with Cy3-CPT and the cDNA of A549-P cells was labeled with Cy5-CPT as a control. Array hybridization was performed in a CapitalBio BioMixer^TM^ II Hybridization station overnight at a rotation speed of 8 rpm and a temperature of 42°C, and subsequently washed with two consecutive solutions (0.2% sodium dodecyl sulfate, 2X saline-sodium citrate at 42°C for 5 min and 0.2X saline-sodium citrate for 5 min at room temperature). These arrays were scanned with a confocal LuxScan™ scanner and the images obtained were then analyzed using LuxScan 3.0 software (CapitalBio Corporation). The obtained images were analyzed using LuxScan 3.0 (CapitalBio Corporation), which employed the LOWESS normalization method (33). The differentially expressed genes that exhibited an average ratio in triplicate tests >2.0-fold upregulated or <0.5-fold downregulated were obtained. Significance Analysis of Microarrays (LightCycler software version 3.02) was performed.

### Identification of differentially expressed genes by qPCR

To further identify the results of the cDNA microarray data, qPCR was performed in the iCycler iQ5 real-time PCR detection system (Bio-Rad Laboratories) using EQ SYBR-Green dye (Bio-Rad Laboratories) according to the manufacturer’s instructions. The comparative threshold cycle (CT) method was used to calculate the amplification fold. β-actin (forward: 5′-CTTAGTTGCGTTACACCCTTTCTTG-3′ and reverse: 5′-CTTAGTTGCGTTACACCCTTTCTTG-3′) was used as a reference control gene to normalize the expression value of each gene. The primer sequences are listed in Table I.

### Measurement of C3 and C4b concentrations by ELISA

The cells were plated onto 96-well plates at 4.0×10^4^ cells/well with three duplicate wells. Then, the supernatants of each group of cells at 24, 48, 72 and 96 h were respectively collected to measure human complement factor C3 using the C3 ELISA kit (Cusabio, Wuhan, China) and C4b concentration using the C4b ELISA kit (Cusabio) according to the manufacturer’s instructions (34). The protein concentrations of C3 and C4b were quantified in the media supernatants.

### Determination of extracellular phosphate ion concentrations using the phosphomolybdic acid method

The same supernatants of each group of cells at 24, 48, 72 and 96 h were used to detect extracellular phosphate ion concentration by the phosphomolybdic acid method (Nanjing Jiancheng Bioengineering Institute, Nanjing, China) according to the manufacturer’s instructions (35). The quantities of Pi were quantified in the media supernatants.

### Statistical analysis

Each experiment was performed at least three times. Statistical differences between the A549-P-S groups and A549-P groups (or A549 groups) were assessed using one-way analysis of variance and an unpaired Student’s t-test. All analyses were performed using SPSS software, version 19.0 (IBM, Armonk, NY, USA). P*<*0.05 was considered to indicate a statistically significant difference.

## Results

### Downregulation of *SLC34A2* in A549 and H1299 lung adenocarcinoma cell lines

**Figure 1:**
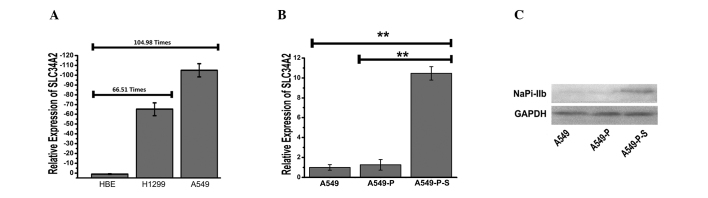
Expression of *SLC34A2* between normal lung cells and lung adenocarcinoma cells and detection of a stable transfection cell line. (A) qPCR analysis was used to assess the mRNA levels of *SLC34A2* expression in A549 and H1299 lung adenocarcinoma cells compared with normal HBE lung cells. (B) mRNA expression levels of *SLC34A2* in the stably transfected cell lines A549, A549-P and A549-P-S were detected by qPCR analysis. (C) Protein expression levels of *SLC34A2* in the stably transfected cell lines A549, A549-P and A549-P-S were determined by western blotting. The values are expressed as the mean ± standard error of the mean of three independent sets of data. ^**^P<0.01. qPCR, quantitative polymerase chain reaction; SLC34A2, solute carrier family 34 (sodium phosphate), member 2.

qPCR was performed to investigate whether the expression of *SLC34A2* was different in A549 and H1299 lung adenocarcinoma cells compared with normal human bronchial HBE cells (Fig. 1A). The results indicated that the expression levels of *SLC34A2* were downregulated 66.51-fold in the H1299 cell line and 104.98-fold in the A549 cell line compared with the HBE cell line (P<0.01). This suggested that *SLC34A2* might be associated with the initiation and progression of lung adenocarcinoma.

### Generation of A549 cells with stably expressing SLC34A2

In order to investigate the effects of *SLC34A2* upregulation in human lung adenocarcinoma, A549 cells stably expressing *SLC34A2* (A549-P-S) or PcDNA3.1 vector (A549-P) were initially selected by G418. qPCR and western blot analysis were performed to detect the expression of *SLC34A2* in A549-P-S compared with A549-P and A549 cells. As shown in Fig. 1B, the expression level of *SLC34A2* in the A549-P-S group was upregulated 10.46-fold compared with the A549 cell line (P<0.01). As shown in Fig. 1C, the *SLC34A2* protein (NaPi-IIb) was identified in the A549-P-S group and was also functionally expressed compared with the A549-P group. The results confirmed that the A549 cell line with stable transfection of *SLC34A2* was constructed successfully.

#### Effects of *SLC34A2* on the viability and invasion of A549 cells

**Figure 2:**
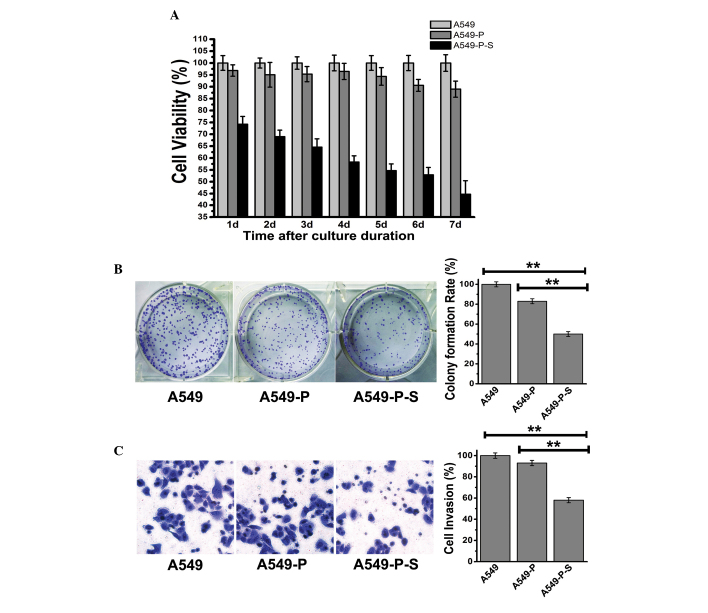
Effects of *SLC34A2* on the viability and invasion of A549 cells. (A) 3-(4,5-dimethylthiazol-2-yl)-2,5-diphenyltetrazolium bromide analysis detected the different cell viability of the stably transfected cell line A549-P-S compared with A549 and A549-P-S cells for seven consecutive days. (B) Colony formation experiment confirmed the different cell growth rate of the stably transfected cell line A549-P-S compared with A549 and A549-P-S cells following 2 weeks. (C) A transwell assay demonstrated the different cell invasion of the stably transfected cell line A549-P-S compared with A549 and A549-P-S following 24 h (magnification, ×200). The values are expressed as the mean ± standard error of the mean of three independent sets of data. ^**^P<0.01. SLC34A2, solute carrier family 34 (sodium phosphate), member 2.

The cell viability of A549, A549-P and A549-P-S cells was examined at consecutive time points (1, 2, 3, 4, 5, 6 and 7 days) using the MTT assay and colony forming assay. As shown in Fig. 2A, the cell viability of the A549-P-S group was the lowest at each time point. As shown in Fig. 2B, the colony formation rate of the A549-P group was 83%; however, it was only 53% in the A549-P-S group (P<0.01). The data were consistent with the results of the growth curve of *SLC34A2*. In addition, a transwell assay was performed to detect the cell invasion ability of *SLC34A2* (Fig. 2C). It was revealed that the quantity of the cells in the chamber decreased by 7% in the A549-P group, however, decreased by 42% in the A549-P-S group (P<0.01). These results indicated that enhancing the expression of *SLC34A2* significantly inhibited the viability and invasion of A549 cells.

### Microarray data analysis and identification of differentially expressed genes

**Figure 3:**
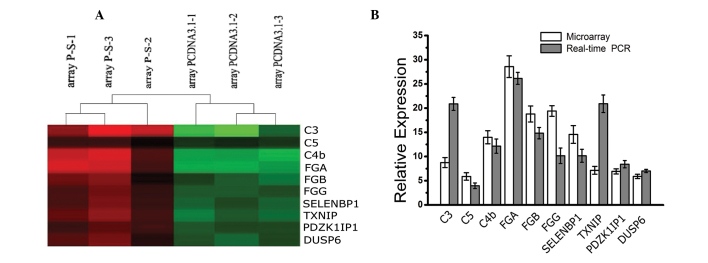
Differentially expressed genes between A549-P-S and A549-P cells by microarray assay and qPCR validation. (A) Each group in triplicate was shown on the same chip. Color intensity was assigned to ratios of gene expression; shades of red indicate genes that were upregulated; shades of green indicate genes that were downregulated. (B) 10 upregulated genes were identified by qPCR, which was consistent with cDNA microarray data. qPCR, quantitative polymerase chain reaction; C3, complement C3 precursor; C4b, complement 4B preproprotein; FGA, fibrinogen α chain precursor; FGB, fibrinogen β chain precursor; FGG, fibrinogen γ chain precursor; SELENBP1, selenium binding protein 1; TXNIP, thioredoxin-interacting protein; PDZK1IP1, PDZK-interacting protein 1; DUSP6, dual specificity protein phosphatase 6.

In order to investigate the molecular mechanisms underlying the effects of *SLC34A2* on the viability and invasion of A549 cells, an oligonucleotide microarray was used to screen differentially expressed genes between A549-P-S and A549-P cells (Fig. 3A). As shown in Table II, the expression levels of 10 genes were higher in the A549-P-S group compared with the A549-P group. The upregulated genes included complement genes (C3, C4b and C5), complement-associated genes [fibrinogen α chain precursor (FGA), fibrinogen β chain precursor (FGB) and fibrinogen γ chain precursor (FGG)] and tumor suppressor genes [selenium binding protein 1 (SELENBP1), thioredoxin-interacting protein (TXNIP), PDZK1-interacting protein 1 (PDZK1IP) and dual specificity protein phosphatase 6 (DUSP6)]. To further validate the precision of microarray data, qPCR was performed using the same RNA sample as that used in the microarray. As shown in Fig. 3B, the results of qPCR were marginally different to the microarray; however, the upregulation observed was consistent with the microarray. Therefore, the results suggest that the effects of *SLC34A2* on A549 cells might be associated with alterations in these genes.

### Effect of SLC34A2 on C3 and C4b production in A549 cells

**Figure 4:**
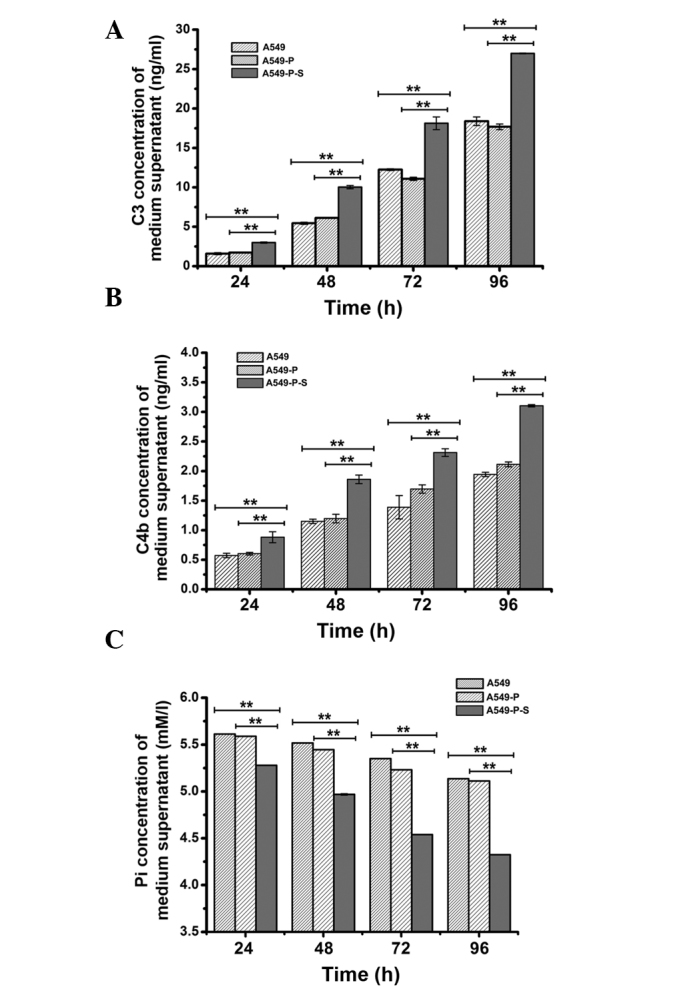
Effects of *SLC34A2* on C3, C4b production and Pi absorption of A549 cells. (A) Effect of *SLC34A2* on C3 production of A549 cells was detected by ELISA assay at consecutive time points. (B) Effect of *SLC34A2* on the C4b production of A549 cells was detected by an ELISA assay at consecutive time points. (C) Effect of *SLC34A2* on the absorption of Pi in A549 cells was assayed by the phosphomolybdic acid method at consecutive time points. The values are expressed as the mean ± standard error of the mean of three independent sets of data. ^**^P*<*0.01. C3, complement C3 precursor; C4b, complement 4B preproprotein; SLC34A2, solute carrier family 34 (sodium phosphate), member 2.

To further evaluate whether enhancing the expression of *SLC34A2* was able to increase the secretion of C3 and C4b in A549 cells, cell culture supernatants of A549, A549-P and A549-P-S cells were used to detect the concentration of C3 and C4b by ELISA at consecutive time points (24, 48, 72 and 96 h). As shown in Fig. 4A, the C3 concentration in cell culture supernatants of the A549-P-S group respectively increased to 1.40 ng/ml (1.26 ng/ml), 4.58 ng/ml (3.90 ng/ml), 5.87 ng/ml (7.24 ng/ml) and 8.59 ng/ml (9.30 ng/ml) at the continuous time points compared with the A549 group (A549-P group; P<0.01). Simultaneously, the other results, shown in Fig. 4B, demonstrated that C4b concentration in the cell culture supernatants of the A549-P-S group respectively increased to 0.30 ng/ml (0.27 ng/ml), 0.71 ng/ml (0.66 ng/ml), 0.92 ng/ml (0.61 ng/ml), 1.16 ng/ml (0.99 ng/ml) at the continuous time points compared with the A549 group (A549-P group; P<0.01). These data further indicated that enhancing the expression of *SLC34A2* was able to significantly increase the secretion of the complement factor C3 and C4b in A549 cells.

### Effect of SLC34A2 on Pi absorption in A549 cells

To determine whether enhancing the expression of *SLC34A2* in A549 cells was able to increase the absorption of Pi in A549 cells and whether increasing Pi absorption was associated with the effect of *SLC34A2* on the secretion of C3 and C4b, cell culture supernatants used in the detection of C3 and C4b concentration were also used to detect Pi concentration using the phosphomolybdic acid method. As shown in Fig. 4C, the Pi concentration in the cell culture supernatants of the A549-P-S group decreased to 0.33 mmol/l (0.31 mmol/l), 0.54 mmol/l (0.47 mmol/l), 0.811 mmol/l (0.69 mmol/l) and 0.812 mmol/l (0.78 mmol/l), respectively, at the continuous time points compared with the A549 group (A549-P group; P<0.01). The Pi concentration and the secretion of C3 and C4b in the A549-P-S group was greater than in the other two groups. The results suggested that not only was enhancing the expression of *SLC34A2* in A549 cells able to increase Pi absorption of A549 cells, but also enhancing Pi absorption capacity may have a certain connection with the effect of *SLC34A2* on the secretion of C3 and C4b in A549 cells.

## Discussion

The role of *SLC34A2* in carcinogenesis has not been fully elucidated, however, previous studies have indicated that this gene might be a potential molecular marker in carcinogenesis. Gaiłza *et al* (36) found that the expression of *SLC34A2* was increased in papillary thyroid carcinoma, and suggested that this gene might be used as a potential biomarker in the diagnosis of papillary thyroid carcinoma. Blanchard *et al* (37) revealed that the expression of *SLC34A2* was decreased in breast cancer, suggesting that this gene had a certain association with breast cancer. Yin *et al* (38) found that *SLC34A2* was able to be used as a target for MX35 to treat ovarian cancer. Previous studies have demonstrated that the expression of *SLC34A2* between normal lung tissue and lung cancer tissue was significantly different (12). To the best of our knowledge, the present study provided the first evidence, that the expression levels of the *SLC34A2* gene in A549 and H1299 cells are clearly different compared with HBE cells by qPCR. Furthermore, the results demonstrate that enhancing the expression of *SLC34A2* in A549 cells was able to significantly suppress the viability and invasion of A549 cells *in vitro*. These results imply that downregulation of *SLC34A2* may be associated with the initiation and progression of lung adenocarcinoma.

Microarray analysis is able to simultaneously analyze almost 10,000 genes in a chip with added benefits, including high-flux and high-sensitivity (39). For further examining the potential mechanisms of *SLC34A2* in lung adenocarcinoma, differentially expressed genes between A549-P and A549-P-S cells were screened using microarray analysis. A total of 10 upregulated genes, including complement genes (C3, C4b and C5), complement associated genes (FGA, FGB and FGG) and tumor suppressor genes (SELENBP1, TXNIP, PDZK1IP1 and DUSP6) were identified by microarray and qPCR. The results suggested that the effects of SLC34A2 on A549 cells might be associated with the expression changes of these genes.

AT II cells possess immune functions. They can directly synthesize factors of immune regulation, including the complement C2, C3, C4 and C5 factors and interleukin (IL)-3 (40). It is well known that the complement system (C1–C9) is important in immunosurveillance in the initial stage of tumorigenesis (41). Usually, the antigen-antibody complex formed through an antibody combining with a tumor cell antigen is able to activate C1 and C4, thereby further activating C3, and then activating a complement cascade to kill tumor cells *in vivo* (42). Another study indicated that the tumor cells themselves could also directly activate complement C3, resulting in activation of the complement alternative pathway to kill tumor cells (43). In addition, the complement alternative pathway was also activated when complement C3 bound to the tumor cell receptor *in vitro*, resulting in the formation of the membrane attack complex (C5b-9) to destroy the tumor cell membrane lipid bilayer and cause the tumor cell to be lysed (44). As the key to activating the complement alternative pathway, C3 is the most important complement factor in the complement alternative pathway. In addition, C4b is an active fragment of C4, which is involved in activating C3 (43,44). C5 is a key complement factor involved in the formation of the membrane attack complex. The present study revealed that enhancing the expression of *SLC34A2* was able to increase mRNA expression levels of C3, C4b and C5 in A549 cells by microarray analysis. Furthermore, the present study confirmed that enhancing the expression of *SLC34A2* was also able to stimulate the secretion of C3 and C4b in A549 cells by an ELISA assay. Rothman *et al* (45,46) found that cytokines IL-1, IL-2 as well as lipopolysaccharide increased C3 production in A549 cells. In addition, dexamethasone and interferon-γ had the same effect on A549 cells (34). Duerst *et al* (47) found that monoclonal antibodies (mAbs) were able to activate C3 to induce complement-dependent cytotoxicity, which led to tumor cells lysis. With the exception of mAbs, corresponding inhibitors of membrane complement regulatory proteins were also able to activate the complement alternative pathway through activating C3 (48). However, at present, there is no report of a relevant gene that is capable of activating C3 production *in vitro*. To the best of our knowledge, the present study was the first to reveal that *SLC34A2* was able to increase the secretion of C3 and C4b in A549 cells.

In AT II cells, *SLC34A2* is responsible for the synthesis of phosphate, which is the main component of surfactant in AT II cells. The surfactant is able to increase the activity of membrane proteins and the mobility of phospholipids (49). Previous studies found that improving the activity of cell surface proteins was useful for activating C3 to trigger the complement alternative pathway (50,51). The present study confirmed that enhancing the expression of *SLC34A2* was able to significantly strengthen the ability to absorb Pi in the A549 cell line. The results of the present study also demonstrated that the effect of *SLC34A2* on promoting the secretion of C3 and C4b may be associated with a time-dependent increase of Pi absorption. Based on the these results, it was hypothesized that the effect of the inhibition of *SLC34A2* on the viability and invasion of A549 cells *in vitro* might be attributed to the activation of the complement alternative pathway by C3 activation and increased Pi absorption. It was also suggested that the lack of Pi might aid the escape of abnormal AT II cells from complement-associated immunosurveillance in the initial stages of lung adenocarcinoma development, when downregulation of *SLC34A2* induces aberrant Pi transport. Therefore, downregulation of *SLC34A2* might cause abnormal AT II cells to develop into lung adenocarcinoma cells. However, in the future, investigation of the effects of inhibiting C3 and C4b on the proliferation of lung cancer cells is required.

Adequate phosphate absorption is important for the maintenance of cellular metabolism (52). The study by Xu *et al* (30) demonstrated that the low Pi environment was able to activate certain cell signaling pathways associated with tumorigenesis in the normal lung cells, including AKT signaling pathways. AKT signaling pathways were generally active in lung cancer cells (53). A total of four tumor suppressor genes were identified including, SELENBP1, TXNIP, PDZK1IP1 and DUSP, which were upregulated in A549-P-S cells compared with A549-P cells. *DUSP6* is an upstream inhibitor of ERK2, which is an important signaling protein in the AKT signaling pathway. Overexpression of *DUSP6* in NSCLC may inactivate ERK2 and further act as a natural terminator of AKT/MAPK signal transduction (54). The present study found that increasing the expression of *SLC34A2* in A549 cells was able to increase DUSP6 expression. Furthermore, Pi absorption in A549 cells increased. Therefore, it was hypothesized that the effects of *SLC34A2* on the viability and invasion of A549 cells might be associated with the upregulation of DUSP6 in the AKT/ERK2 signaling pathway. However, further investigation is in progress. As a member of the selenium-containing protein family, the expression of *SELENBP1* is commonly decreased in numerous types of human epithelial cancer, and this decrease is correlated with a poor prognosis. Previous studies have suggested that SELENBP1 exerts a tumor suppressor function by inhibition of proliferation and was identified as a lung cancer suppressor HIF-1 target gene (55–58). TXNIP is a member of the thioredoxin pathway and a tumor metastasis suppressor gene (59). PDZK1IP1 exhibited a tumor-suppressor phenotype in cultured colon cancer cells by negatively affecting proliferation and tumor growth (60). Therefore, our data suggested that a low Pi environment induced by downregulation of *SLC34A2* in A549 cells may cause expression changes of these genes associated with tumorigenesis signaling pathways, resulting in AT II cells that develop into lung adenocarcinoma cells.

The present study first determined that the expression levels of *SLC34A2* were downregulated in A549 and H1299 lung adenocarcinoma cells, then further revealed that the elevated expression of *SLC34A2* was able to significantly inhibit the viability and invasion of A549 cells *in vitro*. These results indicated that *SLC34A2* may be important in the initiation and progression of lung adenocarcinoma. The present study also indicated that the relative mechanisms of *SLC34A2* in A549 lung cancer may be associated with the activation of the complement alternative pathway (C3 and C4b) and upregulation of the expression of SELENBP1, TXNIP, PDZK1IP1 and DUSP6*.* These incidents might be attributed to enhancing Pi transport in A549 cells by elevating the expression of *SLC34A2*. From this it was hypothesized that the downregulation of *SLC34A2,* expressed primarily in AT II cells, might cause abnormal AT II cells to escape from complement-associated immunosurveillance and abnormally express certain tumor-suppressor genes, inducing development of these cells into lung adenocarcinoma. The present study has provided further insights into the effects and mechanisms of *SLC34A2* in lung cancer.

## Figures and Tables

**Table I:** Primer sequences and detection results of differentially expressed genes identified by qPCR.

Gene name	Primer direction	Primer sequence (5′-3′)	Product (bp)	Upregulation fold
C3	F-1	AAAGATAAGAACCGCTGGGAGG	117	20.88
	R-1	CACGACGGGAGGCACAAA		
C5	F-1	CCCACTACAGAGGCTACG	305	3.18
	R-1	AGGACTGAGAAACCCAAC		
C4b	F-1	CTGTCTGCCTACTGGATTGC	205	12.15
	R-1	CCTTCAGGGTTCCTTTGC		
FGA	F-1	AGGCAACACTTACCACTG	176	26.15
	R-1	GTAATCTCATTTCCACCAG		
FGG	F-1	ACATTGCCAATAAGGGAG	209	10.15
	R-1	TGTTGTGCCAGTAGGAGA		
FGB	F-1	TTGCCCATAGAAACGAGG	153	14.82
	R-1	GTGGCGACTTGGAGTGAA		
SELENBP1	F-1	CAAAGTATGGCTACAGGG	84	10.16
	R-1	AGTGGCTCTAAGACGATT		
TXNIP	F-1	CCACCGTCATTTCTAACT	147	20.90
	R-1	ACACCTCCACTATCACCC		
PDZK1IP1	F-1	TTTCAGGCGGACACCAAT	217	8.41
	R-1	ACGAGGACCAGGAACACG		
DUSP6	F-1	AGCGACTGGAACGAGAAT	297	7.01
	R-1	GTTGGACAGCGGACTACC		

F, forward; R, reverse; C3, complement C3 precursor; C5, complement C5 precursor; C4b, complement 4B preproprotein; FGA, fibrinogen α chain precursor; FGG, fibrinogen γ chain precursor; FGB, fibrinogen β chain precursor; SELENBP1, selenium binding protein 1; TXNIP, thioredoxin-interacting protein; PDZK1IP1, PDZK1-interacting protein 1; DUSP6, dual specificity protein phosphatase 6.

**Table II:** Microarray analysis to determing overexpressed genes in an A549 cell line stably expressing *SLC34A2*.

GenBank acc. no.	Average ratio (Cy5/Cy3)	GenBank identity	Gene ontology molecular function
NM_001002029	13.98	*Homo sapiens* complement component 4B preproprotein (C4B)	Complement and coagulation cascades
NM_000064	8.75	*Homo sapiens* complement C3 precursor (C3)	Complement and coagulation cascades
NM_001735	5.88	*Homo sapiens* complement C5 precursor (C5)	Complement and coagulation cascades
NM_021871	28.58	*Homo sapiens* fibrinogen α chain precursor (FGA)	Complement and coagulation cascades
NM_000509	19.41	*Homo sapiens* fibrinogen γ chain precursor (FGG)	Complement and coagulation cascades
NM_005141	18.80	*Homo sapiens* fibrinogen β chain precursor (FGB)	Complement and coagulation cascades
NM_005025	14.56	*Homo sapiens* selenium binding protein 1 (SELENBP1)	Contributed - metabolic_process--Hs_Selenoproteins
NM_006472	7.15	*Homo sapiens* Thioredoxin-interacting protein (TXNIP)	Tumor suppressor and thioredoxin pathway
NM_005764	6.93	*Homo sapiens* PDZK1-interacting protein 1 (PDZK1IP1)	Tumor suppressor
NM_001946	5.90	*Homo sapiens* dual specificity protein phosphatase 6 (DUSP6)	Activates extracellular signal-regulated kinase 2 (ERK2)

SLC34A2, solute carrier family 34 (sodium phosphate), member 2.

## Abbreviations

SLC34A2: solute carrier family 34 (sodium phosphate), member 2
NSCLC: non-small cell lung cancer
CAV1: caveolin 1
C3: complement C3 precursor
C4b: complement 4B preproprotein
C5: complement C5 precursor
FGA: fibrinogen α chain precursor
FGB: fibrinogen β chain precursor
FGG: fibrinogen γ chain precursor
SELENBP1: selenium binding protein 1
TXNIP: thioredoxin-interacting protein
PDZK1IP1: PDZK1-interacting protein 1
DUSP6: dual specificity protein phosphatase 6
MTT: 3-(4,5-dimethylthiazol-2-yl)-2,5-diphenyltetrazolium bromide
qPCR: quantitative polymerase chain reaction
AT II: alveolar type II
mAbs: monoclonal antibodies
OD: optical density
